# Effects of Postoperative Pain Management on Immune Function After Laparoscopic Resection of Colorectal Cancer: A Randomized Study

**DOI:** 10.1097/MD.0000000000003602

**Published:** 2016-05-13

**Authors:** So Yeon Kim, Nam Kyu Kim, Seung Hyuk Baik, Byung Soh Min, Hyuk Hur, Jinae Lee, Hyun-young Noh, Jong Ho Lee, Bon-Neyo Koo

**Affiliations:** From the Department of Anesthesiology and Pain Medicine, Anesthesia and Pain Research Institute, Yonsei University College of Medicine (SYK, H-YN, B-NK); Department of Surgery, Division of Colon and Rectal Surgery, Yonsei University College of Medicine (NKK, SHB, BSM, HH); Biostatistics Collaboration Unit, Yonsei University College of Medicine (JL); and Research Center for Silver Science, Institute of Symbiotic Life-TECH, National Leading Research Laboratory of Clinical Nutrigenetics/Nutrigenomics, Department of Food and Nutrition, Brain Korea 21 PLUS Project, College of Human Ecology, Yonsei University (JHL), Seoul, Republic of Korea.

## Abstract

There has been a rising interest in the possible association between perioperative opioid use and postoperative outcomes in cancer patients. Continuous surgical wound infiltration with local anesthetics is a nonopioid analgesic technique that can be used as a postoperative pain management alternative to opioid-based intravenous patient-controlled analgesia (IV PCA). The aim of this study was to compare the effects of an opioid-based analgesic regimen versus a local anesthetic wound infiltration-based analgesic regimen on immune modulation and short-term cancer recurrence or metastasis in patients undergoing laparoscopic resection of colorectal cancer.

Sixty patients undergoing laparoscopic resection of colorectal cancer were randomly assigned to either the opioid group or the ON-Q group. For postoperative analgesia during the first 48 hours, the opioid group (n = 30) received fentanyl via IV PCA, whereas the ON-Q group (n = 30) received continuous wound infiltration of 0.5% ropivacaine with an ON-Q pump and tramadol via IV PCA. Pethidine for the opioid group and ketorolac or propacetamol for the ON-Q group were used as rescue analgesics. Anesthesia was induced and maintained with propofol and remifentanil. The primary outcome was postoperative immune function assessed by natural killer cell cytotoxicity (NKCC) and interleukin-2. Secondary outcomes were postoperative complications, cancer recurrence, or metastasis within 1 year after surgery, and postoperative inflammatory responses measured by white blood cell count, neutrophil percentage, and C-reactive protein. Immune function and inflammatory responses were measured before surgery and 24 and 48 hours after surgery.

Fifty-nine patients completed the study. In the circumstance of similar pain control efficacy between the opioid group and the ON-Q group, postoperative NKCC and interleukin-2 levels did not differ between the 2 groups. The incidence of postoperative complications and recurrence or metastasis within 1 year after surgery was comparable between the groups. Postoperative inflammatory responses were also similar between the groups.

When compared with ropivacaine wound infiltration-based analgesia, fentanyl-based analgesia did not further decrease NKCC or affect short-term cancer recurrence or metastasis. Thus, a fentanyl-based analgesic regimen and a ropivacaine wound infiltration-based analgesic regimen can both be used for postoperative pain management in laparoscopic resection of colorectal cancer.

## INTRODUCTION

Opioids are a common first-choice analgesic for postoperative pain management. Opioid-based intravenous patient-controlled analgesia (IV PCA) has been widely used for postoperative analgesia due to its effectiveness and convenience since the late 1960s^1^; however, there is an emerging interest in the possible association between perioperative use of opioids and recurrence or metastasis after cancer surgery.^2–5^ Immunomodulation by opioids occurs through direct action on immune cells, modulation of the hypothalamic-pituitary-adrenal axis, and modulation of sympathetic activity.^6^ However, the evidence for opioid-induced immunomodulation is conflicting in experimental and human studies. Fentanyl suppresses natural killer (NK) cell function, which plays a major role in innate and adaptive immunity, and increases the risk of tumor metastasis in a rat model.^7,8^ Fentanyl also suppresses postoperative NK cell function in patients.^9^ However, fentanyl can improve NK cell function, and low-dose remifentanil does not impair NK cell function in healthy humans.^10,11^ Likewise, retrospective results regarding the use of postoperative opioids and cancer recurrence remain controversial. Postoperative opioid analgesia was associated with increased risk of cancer recurrence after radical prostatectomy when compared with epidural analgesia.^2^ On the contrary, there was no significant difference in overall or disease-free survival between postoperative opioid analgesia and epidural analgesia after laparoscopic resection of colorectal cancer.^3^

Laparoscopic colorectal surgery has become popular for the surgical treatment of colorectal cancer because there is minimal surgical trauma, less postoperative pain, and a rapid return to preoperative activity levels with a shorter hospitalization period.^12^ It also has shown beneficial outcomes in terms of postoperative immune function, morbidity, cancer recurrence, and cancer-related survival compared with open surgery.^13,14^ Continuous wound infiltration with local anesthetics has been recognized as a useful nonopioid analgesic technique for postoperative pain management after laparoscopic and open colorectal surgeries.^15^ Such nonopioid analgesic techniques can be an alternative choice for cancer patients undergoing surgery, since they can avoid the immunosuppressive effects of opioids. However, no prospective data are available regarding the effects of postoperative pain management with opioids or with continuous wound infiltration on postoperative immune function.

The aims of this randomized study were to compare postoperative immune function assessed by NK cell cytotoxicity (NKCC) and interleukin (IL)-2, which is required for proliferation and cytotoxic activities of NK cells; and to evaluate short-term cancer recurrence or metastasis based on postoperative pain management with opioids or with continuous wound infiltration in patients undergoing laparoscopic resection of colorectal cancer.

## METHODS

This study was approved by the Severance Hospital Institutional Review Board (protocol number: 4–2013–0044) and was registered at http://clinicaltrials.gov (registration number NCT02012244). Between January 2014 and December 2014, we enrolled 60 patients aged 20 to 80 years with an American Society of Anesthesiologists (ASA) physical status I to III who underwent laparoscopic resection of colorectal cancer. These patients were followed until 1 year after surgery. Written informed consent was obtained from every patient. Patients were not admitted to the study if they had drug allergies, significant renal or hepatic impairment, high levels of C-reactive protein (CRP), or leukocytosis (>11,000/μL) before surgery.

### Interventions

Patients were randomly assigned to one of the 2 groups using a random number generator. The opioid group (n = 30) received fentanyl via IV PCA. The ON-Q group (n = 30) received continuous surgical wound infiltration of 0.5% ropivacaine with an ON-Q pump. The ON-Q group also received IV PCA with tramadol to match pain control efficacy between the 2 groups. The fentanyl PCA was composed of 2000 μg fentanyl (Hana Pharm, Seoul, Korea) and 0.3 mg ramosetron (Nasea, Astellas, Tokyo, Japan) mixed with normal saline to a total volume of 200 mL. The tramadol PCA was composed of 450 mg tramadol (Tridol, Yuhan Corp., Seoul, Korea) mixed with normal saline to a total volume of 100 mL. The bolus dose was 1 mL at a basal infusion rate of 1 mL/h, with a lockout interval of 7 minutes in both groups. We used a PCA device (Accumate 1100, WooYoung Medical, Seoul, Korea) in which delivered amounts of the drug were automatically recorded every 30 minutes, and these data were transferred to a computer for analysis. Continuous wound infiltration in the ON-Q group was delivered by an elastomeric pump (ON-Q PainBuster, I-Flow Corp., Lake Forest, CA) through 2 multiholed Soaker catheters, each of which was located between subcutaneous fat and fascia and below the fascia. The ON-Q was composed of 200 mL 0.75% ropivacaine (Naropin, AstraZeneca, Seoul, Korea) and 100 mL normal saline, and was delivered at a flow rate of 4 mL/h (2 mL/h per catheter).

Upon arrival in the operating room, electrocardiogram, pulse oxygen saturation, invasive arterial pressure, and bispectral index (BIS; A-2000 SP, Aspect Medical Systems, Norwood, MA) were determined. Anesthesia was induced and maintained with propofol (Fresofol, Fresenius Kabi Korea Ltd, Seoul, Korea) and remifentanil (Ultiva, GlaxoSmithKline, Brentford, UK) using a target-controlled infusion by a commercial total IV anesthesia pump (Orchestra Base Primea, Fresenius-Vial, Sévres, France). The depth of anesthesia was maintained at a BIS value of 40 to 60. Propofol and remifentanil were chosen as anesthetic agents because neither impairs NK cell activity.^11,16^ Controlled ventilation was performed with an 8 mL/kg tidal volume and a positive end-expiratory pressure of 5 cm H_2_O, and ventilator frequency was adjusted to maintain an end-tidal carbon dioxide (CO_2_) between 35 and 40 mm Hg. Pneumoperitoneum was induced by insufflation of CO_2_, and the intra-abdominal pressure was maintained at 12 to 15 mm Hg. At 10 minutes before the end of surgery, 50 μg fentanyl in the opioid group and 50 mg tramadol in the ON-Q group were given, and 0.3 mg ramosetron was given in both groups. In the ON-Q group, a 5-mL bolus of 0.75% ropivacaine was injected through each catheter of the ON-Q pump at the end of surgery. IV PCA with fentanyl or tramadol was initiated at the end of surgery and maintained during postoperative 48 hours in each group.

### Data Collection

The primary outcome of this study was postoperative immune function assessed by NKCC and IL-2. Secondary outcomes were postoperative complications and cancer recurrence or metastasis within 1 year after surgery, and postoperative inflammatory responses measured by white blood cell (WBC) count, neutrophil percentage, and CRP. Immune function and inflammatory responses were measured before the surgery, and 24 and 48 hours after surgery. Postoperative complications were assessed by the Postoperative Morbidity Survey, which is a reliable and valid survey of postoperative morbidity in major elective surgery.^17^ Cancer recurrence or metastasis was checked with a colonoscopy or computed tomography.

Because of the possible impact of pain intensity on postoperative immune function, we checked pain intensity using an 11-point numerical rating scale (NRS), from 0 to 10 (0 = no pain and 10 = worst pain) at rest and while coughing 1, 6, 12, 24, and 48 hours postoperatively and controlled pain with a target of resting NRS <4 using rescue analgesics (pethidine for the opioids group and ketorolac or propacetamol for the ON-Q group). In addition, the patients were advised to press the PCA bolus button when NRS ≥4.

### Assay for NKCC

Isolation of peripheral blood mononuclear cells and the NKCC assay was performed as previously described.^18^ After whole blood was mixed with the same volume of Roswell Park Memorial Institute (RPMI) 1640 medium (Gibco, Invitrogen, CO), it was laid on a Histipaque-1077 (Sigma, CA) and centrifuged (2000 rpm for 20 minutes at 10°C). Then, a thin layer of peripheral blood mononuclear cells was harvested and washed twice with RPMI 1640 and resuspended in RPMI 1640 containing streptomycin.

NKCC was determined with the CytoTox 96 Non-Radioactive Cytotoxicity Assay (Promega Co., WI) using K562 cells as the target cell line. This colorimetric assay quantitatively measures lactate dehydrogenase (LDH), a stable cytosolic enzyme that is released upon cell lysis, in much the same way that ^51^Cr is released in a radioactive assay. Briefly, peripheral blood mononuclear cells (effector cell, E) and K562 cells (2 × 10^4^ cells/well; targeted cell, T) were mixed in different E:T ratios (10:1, 5:1, and 2.5:1) in a 96-well and incubated at 37°C with 5% CO_2_ overnight according to the manufacturer's instructions.^19^ The NKCC of effector cells was measured with a 2030 multilabel reader (Victor X5, PerkinElmer) at 490 nm and was calculated using the following equation: 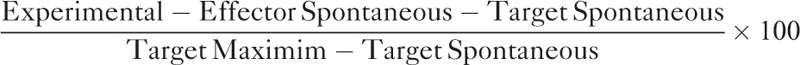


“Experimental” is the experimental LDH release of cocultured effector and target cells, “effector spontaneous” and “target spontaneous” express the spontaneous released LDH of the effector and target cells alone, respectively, and “target maximum” is the maximum LDH release of target cells.

### Interleukin-2 Assay

Interleukin-2 was measured in serum using a commercial ELISA kit (Quantikine Human IL-2 ELISA kit; R&D System Inc.) The absorbance was read at 450 nm using Spectra Max 190 micro-plate reader (Molecular Devices, Sunnyvale, CA).

### Statistical Analysis

In previous studies that demonstrated the effect of fentanyl and remifentanil on NKCC in normal volunteers, 7 and 10 subjects were enrolled, respectively.^10,11^ About 30 subjects in each group can reveal the different effects of drug therapy on NKCC.^18^ Therefore, we decided to include 30 patients in each group.

All data are presented as mean ± SD or median (interquartile range [IQR]) for continuous variables or the number of patients (percentage) for categorical variables. The Shapiro–Wilk test and Q–Q plot were used to test continuous variables for the normality assumption. Normally distributed variables were analyzed with the independent *t* test, and non-normally distributed variables were analyzed with the Mann–Whitney *U* test. Categorical variables were evaluated with the chi-square test or Fisher exact test, as appropriate. Variables measured over time were analyzed with a linear mixed model, and the Mann–Whitney *U* test and Wilcoxon signed-rank test were used to test variables that did not meet normality. Adjustment for multiple comparisons was performed with the Bonferroni correction method. Two-sided *P* values of <0.05 were considered to indicate statistical significance. Statistical analyses were performed using SAS version 9.2 (SAS Inc., Cary, NC).

## RESULTS

**FIGURE 1:**
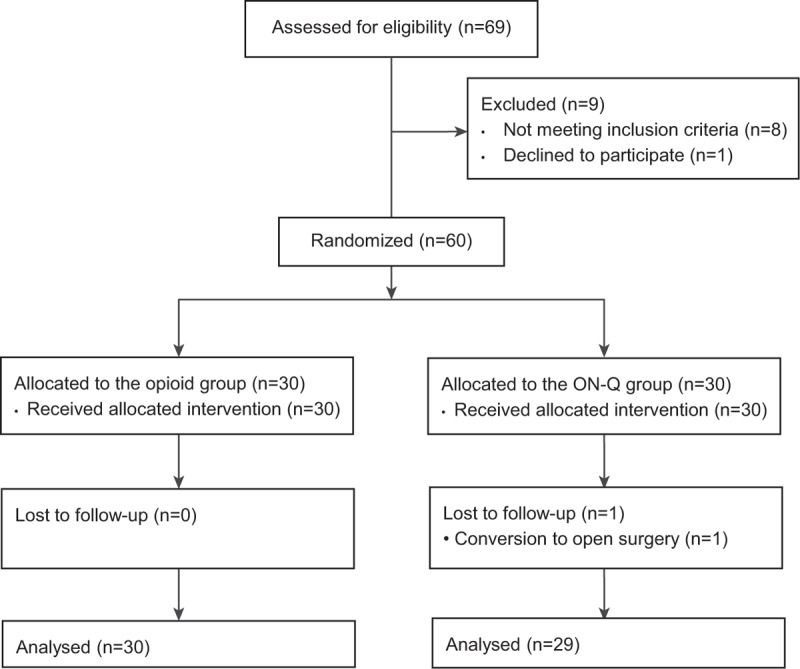
Patient assignment to study groups (randomized) and treatment protocols.

Of the 69 patients assessed for eligibility, 60 patients were enrolled and randomly assigned to the groups and 59 patients (98 %) completed the study (Figure 1). One patient was excluded from analysis due to conversion to open surgery. There were no significant differences in the patient characteristics including pathologic stage and operation details between the 2 groups (Table 1). The total administered dose of fentanyl was 994 ± 296 μg (mean ± SD) in the opioid group and the dose of tramadol was 376 ± 71 mg (mean ± SD) in the ON-Q group during the first 48 hours after surgery.

**TABLE 1:**
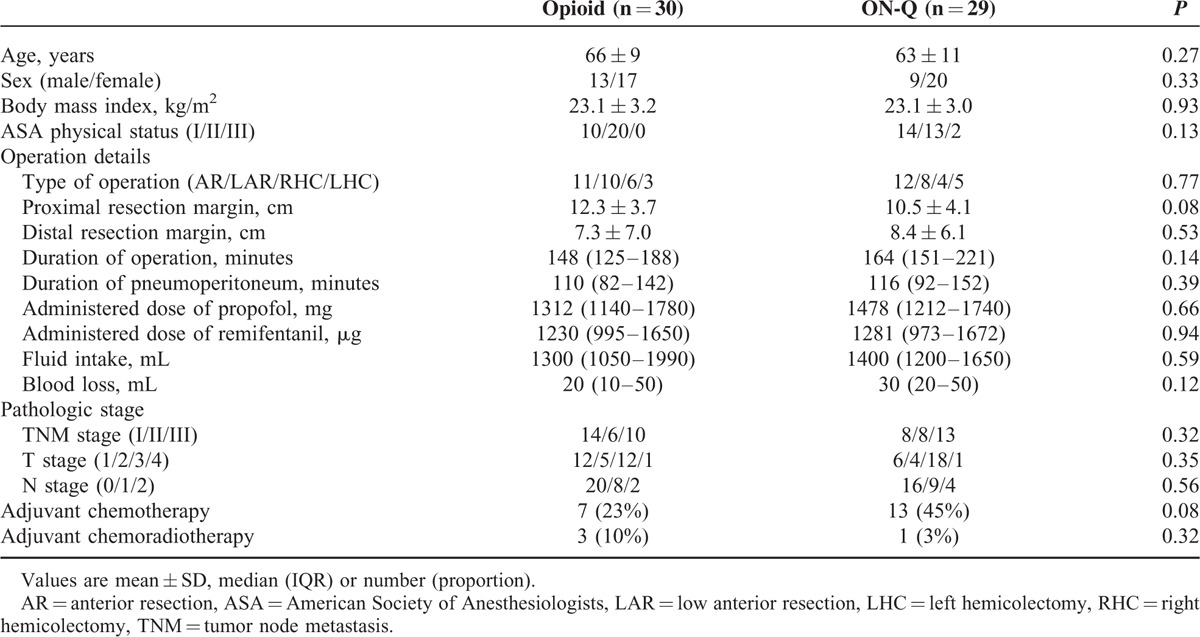
Patient Characteristics

### Postoperative Immune Function

**FIGURE 2:**
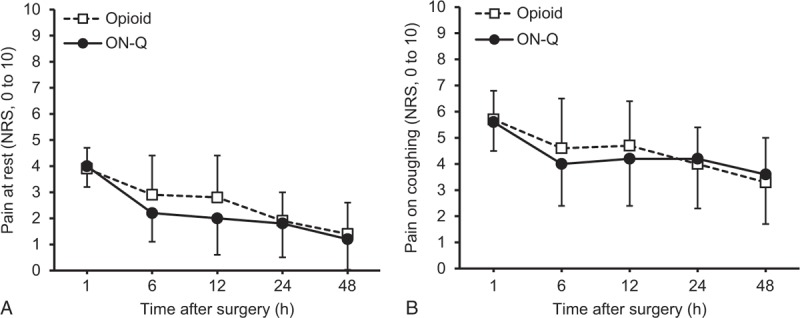
Numerical rating scale (NRS) for pain (A) at rest and (B) while coughing 1, 6, 12, 24, and 48 hours after surgery. Data are expressed as mean ± SD. No difference between the groups.

**FIGURE 3:**
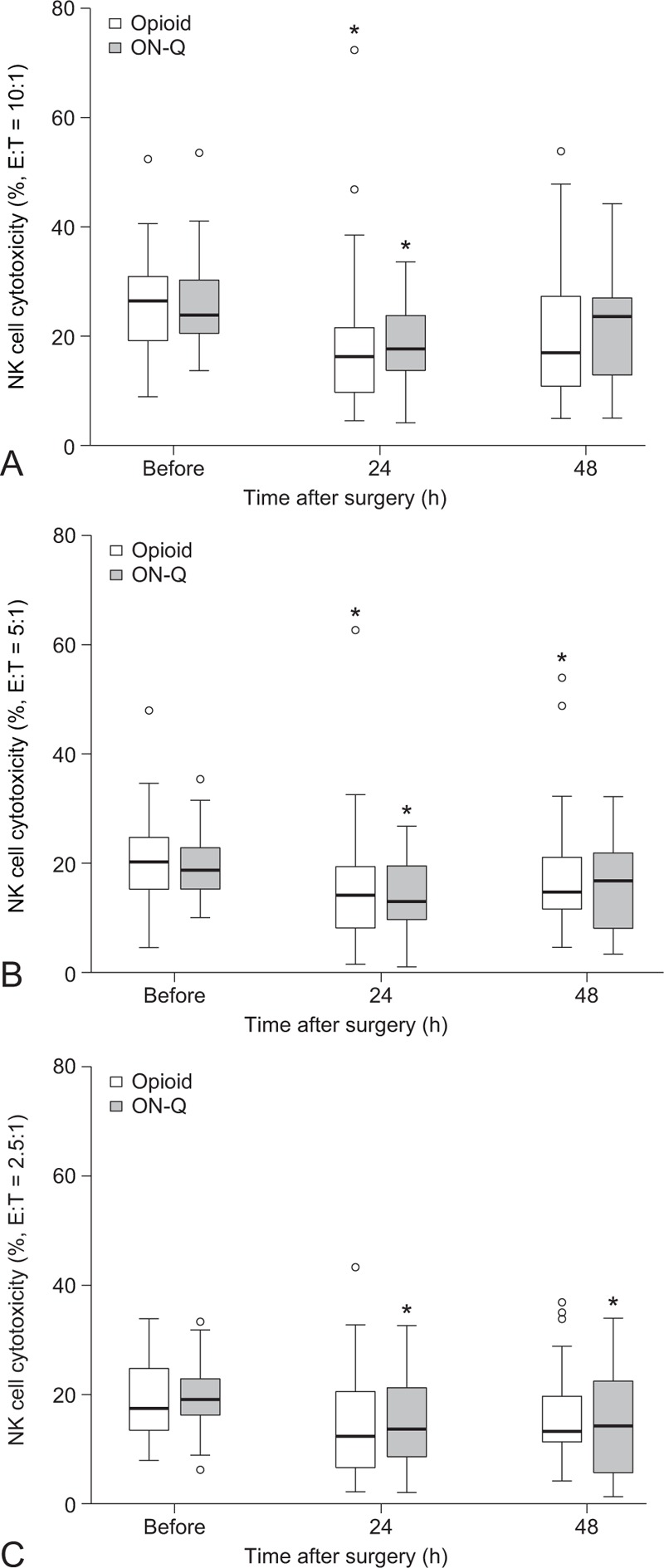
Changes in natural killer cell cytotoxicity after surgery. Box plot indicates the median (solid line), interquartile range (box), and values within 1.5 times the interquartile range (whiskers). Outliers are indicated by circles. No difference between the groups. ^∗^*P* < 0.05 versus “before surgery” for each group (Bonferroni-corrected). E = effector cell, T = target cell.

As the 2 different pain management strategies showed similar pain control efficacy (Figure 2), the possible impact of different pain intensity between the 2 groups on postoperative immune function can be ruled out. Postoperative immune function assessed by NKCC and IL-2 were not different between the 2 groups (Figure 3 and Table 2). NKCC tended to decrease 24 hours after surgery and recover 48 hours after surgery in both groups (Figure 3). Postoperative IL-2 levels significantly increased in both groups compared with preoperative values (Table 2). The difference between preoperative and postoperative NKCC (NKCC 24 or 48 hours after surgery − NKCC before surgery) and the difference between preoperative and postoperative IL-2 (IL-2 24 or 48 hours after surgery − IL-2 before surgery) were also similar between the groups (data not shown).

**TABLE 2:**
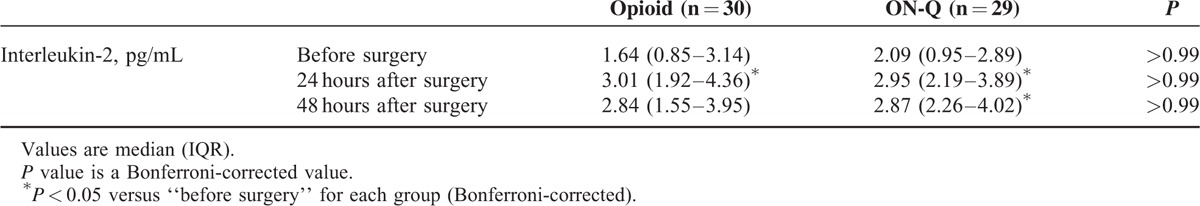
Changes in Interleukin-2 Levels After Surgery

### Postoperative Outcomes

Postoperative hospital stay was 6 (5–8) days in the opioid group and 6 (5–7) days in the ON-Q group (median [IQR], *P* = 0.147). One patient in the opioid group was lost to follow-up before 1 year after surgery. There was no patient mortality. The incidence of postoperative complications and cancer recurrence or metastasis within 1 year after surgery was comparable between the 2 groups. Three patients in the opioid group had complications: postoperative anastomotic leakage 40 days after surgery (colon and small bowel resection with end-to-end anastomosis), intestinal obstruction 10 months after surgery (conservative management), and intestinal obstruction 15 days after surgery (conservative management). Three patients in the ON-Q group had complications: septic shock during chemotherapy 4 months after surgery (conservative management), acute kidney injury 21 days after surgery (conservative management), and intra-abdominal abscess 5 days after surgery (abscess drainage with pig-tail catheter). One patient in the opioid group had liver metastasis 3 months after surgery (wedge resection of the liver), whereas 1 patient in the ON-Q group had lung metastasis 9 months after surgery (chemotherapy).

### Postoperative Inflammatory Responses

There was no difference in the postoperative inflammatory responses assessed by WBC count, neutrophil percentage, and CRP between the 2 groups (Table 3). In each group, postoperative WBC count, neutrophil percentage, and CRP were increased compared with preoperative values (Bonferroni-corrected *P* < 0.001).

**TABLE 3:**
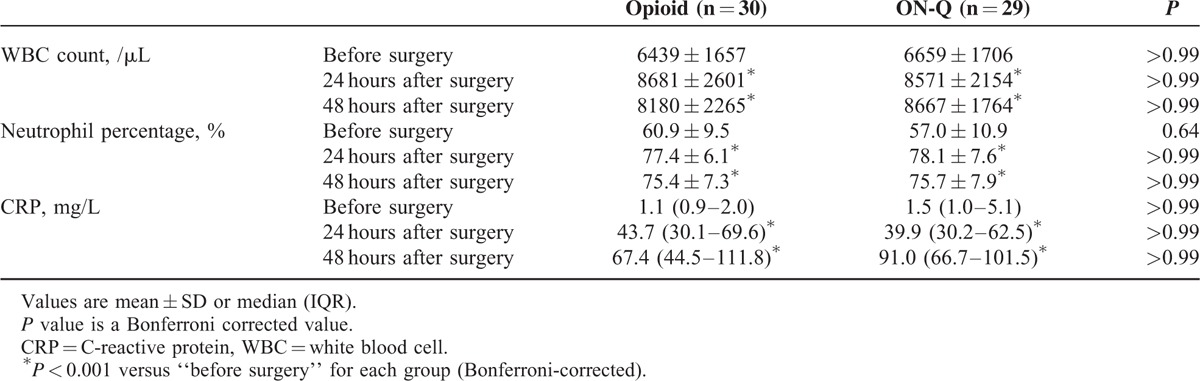
Changes in White Blood Cell Count, Neutrophil Percentage and C-reactive Protein After Surgery

## DISCUSSION

The results of this prospective randomized trial suggest that fentanyl IV PCA and the ropivacaine wound infiltration technique with additional IV tramadol can be used in laparoscopic surgery for colorectal cancer from an immunological standpoint. These 2 pain management methods were comparable in immunomodulation as assessed by NKCC and IL-2 levels, and showed no difference in short-term outcomes. In addition, postoperative inflammatory responses were similar between the 2 pain management methods.

Cancers including colorectal cancer have direct immunosuppressive effects on cell-mediated immunity.^4,5,20^ Even though surgery is the mainstay for solid tumors, surgery itself can promote the development of metastasis by releasing tumor cells into circulation, suppressing the cellular immune system, and augmenting angiogenesis.^4,5^ Therefore, antitumor immune potential can have a significant impact on postoperative outcomes in cancer patients. Some experimental and retrospective clinical evidence indicates an association between anesthetic and analgesic technique and cancer recurrence or metastasis, although prospective, randomized clinical trials are scarce.^2,15,17^

Opioids can affect cell-mediated and humoral immunity by acting directly on immune cells, the hypothalamic-pituitary-adrenal axis, and sympathetic activity.^6^ However, the results of opioid-induced immunomodulation are conflicting in experimental and human studies. In a rat model of breast cancer metastasis, low (100 μg/kg) and high doses (300 μg/kg) of fentanyl administered 2 hours before, at the same time, or 1 hour after tumor inoculation promoted lung tumor retention, which was significantly correlated with the suppression of NKCC by fentanyl.^7^ Administration of fentanyl (40 μg/kg) 1 hour before laparotomy was shown to suppress NKCC and significantly increased the number of lung metastasis compared with clonidine and ketamine in a rat model.^8^ Similarly, in a study with patients undergoing abdominal surgery, half of them with malignant disease and half of them with benign conditions, high-dose fentanyl (75–100 μg/kg) and lower-dose fentanyl (up to 6 μg/kg) during anesthesia had similar effects on the suppression of NKCC 24 hours after abdominal surgery.^9^ This suppression was more prolonged in patients who received a high dose of fentanyl, in which NKCC suppression lasted until 48 hours after surgery. In contrast to the previous stated studies, another study showed that clinically relevant doses of fentanyl (initial does of 3 μg/kg followed by a 2-hour infusion of 1.2 μg/kg/h) significantly increased NKCC at the end of infusion in healthy participants.^10^ In our study, the mean administered dose of fentanyl during the first 48 hours after surgery was 994 μg (minimum 549 μg and maximum 2000 μg), and rescue pethidine (minimum 25 mg and maximum 100 mg) was allowed in 17 patients out of 30 patients in the opioid group. These doses of opioids did not induce additional suppression of NKCC compared with the local anesthetic wound infiltration technique with IV tramadol.

NK cells participate in both innate and adaptive immunity.^21^ They play an important role in immune surveillance against local tumor growth and metastasis by direct cellular cytotoxicity; thus lower NKCC is associated with increased cancer metastasis.^21,22^ Antitumor responses in NKCC are activated by various cytokines, such as IL-1, IL-2, IL-12, IL-15, IL-18, IL-21, and type I interferons.^21,22^ As IL-2-activated human NK cells can effectively kill colon carcinoma cells in vitro,^23^ we investigated IL-2 for this study. NKCC was significantly decreased and IL-2 was significantly increased after surgery in both groups; however, there was no difference in NKCC and IL-2 between the groups. These findings suggest that the 2 pain management methods have similar immunomodulatory effects in laparoscopic colorectal surgery.

There have been many studies on analgesia methods (opioid analgesia or epidural analgesia) and cancer recurrence and survival after surgery, most of which were retrospective analyses.^4,5^ These results differ based on the type of surgery, and even in colorectal cancer, the results are inconsistent.^4,5^ In a retrospective analysis of open colorectal surgery, there was no difference in cancer recurrence between opioid analgesia and epidural analgesia, except for a potential benefit of epidural analgesia in older patients.^24,25^ However, opioid analgesia was associated with a higher mortality rate than epidural analgesia.^25,26^ In a prospective, randomized study with open colorectal surgery, epidural analgesia was associated with improved survival among patients without preoperative metastasis for 1.46 years after surgery when compared with opioid analgesia, though there was no beneficial effect on long-term survival.^27^ Although epidural analgesia has shown some favorable results in open colorectal surgery, another retrospective study reported that there was no difference between epidural analgesia and opioid analgesia in overall survival or disease-free survival 5 years after laparoscopic colorectal cancer surgery.^3^ Therefore, the survival advantage of epidural analgesia may be limited to open colorectal cancer surgery since the immune response is less affected by laparoscopic colorectal resection,^13^ which can affect morbidity, cancer recurrence, and cancer-related survival.^14^ However, no conclusions can be drawn regarding the effects of analgesia methods on cancer outcomes in laparoscopic colorectal surgery because no prospective results are available.

Even though epidural analgesia is one of the key components of enhanced recovery protocols in colorectal surgery,^28^ the benefit of epidural analgesia remains controversial in minimally invasive laparoscopic colorectal surgery.^29–31^ Randomized clinical trials comparing epidural analgesia versus opioid-based IV PCA in laparoscopic colorectal surgery have produced favorable results for opioid-based IV PCA, showing a faster return of bowel function, fewer overall complications, and shorter hospital stays in patients with IV PCA.^30,31^ Patients with epidural analgesia needed more perioperative vasopressor treatment with no difference in postoperative pain scores compared with IV PCA.^31^ Therefore, opioid-based IV PCA may be more appropriate than epidural analgesia in laparoscopic colorectal surgery.

Continuous surgical wound infiltration with local anesthetics can be used as a postoperative pain management alternative to opioid-based IV PCA after laparoscopic colorectal surgery.^15^ In our study, the ON-Q system with 0.5% ropivacaine combined with IV tramadol was chosen as an alternative pain management method to the traditional opioid analgesia. Many in vitro studies have reported antiproliferative or cytotoxic effects of local anesthetics on tumor cells.^4,5^ Ropivacaine reduced in vitro proliferation of mesenchymal stem cells, which are key players in tumor growth, and increased cytotoxicity in a concentration-dependent manner while inhibiting transcription pathways related to neoplasia and metastasis.^32^ Similarly, all local anesthetics including ropivacaine induced concentration-dependent apoptosis and necrosis in T-lymphoma cells in vitro.^33^ Tramadol, which inhibits the reuptake of serotonin and norepinephrine in addition to binding with low affinity to μ-opioid receptors, can stimulate NK cell activity in both rats and humans.^34,35^ In a rat model, the administration of tramadol (20 and 40 mg/kg) before and after laparotomy significantly prevented surgery-induced suppression of NKCC and blocked the enhancement of lung metastasis induced by surgery. In contrast, the administration of 10 mg/kg of morphine did not prevent surgery-induced immunosuppression and was not able to modify the enhancement of lung metastasis after surgery.^34^ These different immunomodulatory effects of morphine and tramadol can also be found in human studies. In 1 study, patients received either 100 mg tramadol or 10 mg morphine immediately after abdominal surgery for uterine carcinoma. NKCC significantly increased in patients treated with tramadol, though there was no significant change of NKCC in patients with morphine.^35^ Considering the favorable impact of tramadol on cellular immunity, tramadol may be a good alternative analgesic to opioids in cancer patients. Contrary to our expectation that ropivacaine wound infiltration with IV tramadol is more beneficial for immune function than opioid analgesia, postoperative NKCC and 1 year cancer recurrence or metastasis were similar between the 2 groups. This may be because this study was performed in patients who received laparoscopic surgery, which induces less immunosuppression than open surgery.^13^

There are some limitations to this study. First, we only measured NKCC during the first 48 hours after surgery and followed up on patients for cancer recurrence or metastasis for 1 year after surgery. In laparoscopic colorectal surgery, IV PCA is usually stopped within 2 days after surgery,^30^ and our center also uses IV PCA for 2 days after surgery. Therefore, we checked NKCC only for 48 hours postoperatively, during which 2 pain management methods were applied. Even though the 1-year rates of cancer recurrence or metastasis were similar between the 2 groups, further studies that evaluate long-term outcomes are needed. Second, we cannot exclude the possibility of immunosuppression by higher doses of fentanyl than used in this study, as the mean administered dose of fentanyl during the first 48 hours after surgery was 994 μg, with a maximum dose of 2000 μg in the opioid group. However, as the patient self-administered fentanyl via the PCA bolus button, there may be no need for higher doses of fentanyl than used in this study for laparoscopic colorectal surgery. In addition, Spearman correlation analysis showed no correlation between the administered dose of fentanyl and postoperative NKCC within the range of fentanyl used in this study. Lastly, we assessed only NK cells as a surrogate marker for immune function, although T-helper cells and cytotoxic T cells are also antitumor effector cells. We chose NK cells since they are involved in both innate and adaptive immunity, unlike T cells, which mediate only adaptive immunity.^21^ Furthermore, NK cells are an emerging target for immunotherapy in cancer patients because of their vital role in immune defense against cancer.^22,23^

In conclusion, fentanyl-based analgesia and ropivacaine wound infiltration-based analgesia have similar immunomodulatory effects after laparoscopic resection of colorectal cancer. As there was no difference in cancer recurrence or metastasis during 1 year after surgery, there is no need to refrain from opioid use as perioperative pain management in laparoscopic colorectal surgery. The ropivacaine wound infiltration technique with additional IV tramadol can be considered as an alternative pain management to opioid-based IV PCA in laparoscopic surgery.
