# Effects of Postoperative Pain Management on Immune Function After Laparoscopic Resection of Colorectal Cancer: A Randomized Study: Erratum

**DOI:** 10.1097/01.md.0000484761.04746.41

**Published:** 2016-06-17

In the article “Effects of Postoperative Pain Management on Immune Function After Laparoscopic Resection of Colorectal Cancer: A Randomized Study”,^1^ which appeared in Volume 95, Issue 19 of *Medicine*, Dr. Koo's name was originally spelled incorrectly. The article has since been corrected online.
